# When Sustainability Meets Innovation: A Cross-Country Study on Dairy Consumer Choices in Poland, Germany, and Czechia

**DOI:** 10.3390/foods15010111

**Published:** 2025-12-30

**Authors:** Ewa Halicka, Małgorzata Kosicka-Gębska, Jerzy Gębski, Krystyna Rejman

**Affiliations:** Institute of Human Nutrition Sciences, Warsaw University of Life Sciences WULS-SGGW, Nowoursynowska 159c, 02-776 Warsaw, Poland

**Keywords:** food choice, innovative dairy, consumer behaviour, sustainable diet, environment, Logistic Regression Model

## Abstract

Consumer food choices play a significant role in supporting sustainable, resilient, and equitable food systems by shaping the environmental, economic, and social impact of diets. To determine whether environmental concerns and innovativeness drive Europeans to buy more sustainable foods, quantitative data were collected from 3131 adults in three countries. A Logistic Regression Model was developed to assess the quantitative impact of variables on consumers’ likelihood to choose sustainably produced foods. Respondents who paid attention to whether food items are produced and/or packaged in an environmentally friendly way were 94% and 48% more likely to purchase sustainably produced products, respectively. Readiness to purchase a dairy product that the buyer had never heard of resulted in a 15% increase in the likelihood of selecting sustainably produced foods. Additionally, respondents living in Germany were 30% more likely to choose sustainable products compared to Polish consumers, while Czech consumers were 10% less likely to do so. Implementing campaigns focusing on promoting sustainable diets could consequently determine and accelerate the adoption of environmentally friendly production practices in the food system. Our findings provide evidence for policymakers, the business community, and educators who aspire to improve the health of people and the planet as a whole.

## 1. Introduction

The dairy sector is a vital component of food systems and milk is one of the biggest and most valuable agricultural commodities in the world. The global total milk output reached 950 million tons in 2024 and is expected to grow steadily, primarily driven by higher yields per animal [1,2]. The estimated number of dairy farms worldwide surpasses 133 million, supporting the livelihoods of 600 million people [3]. In the European Union (EU) milk is produced in all the Member States and represents a significant proportion of the value of the EU agricultural output. Germany, France, Poland, and the Netherlands account for approximately 70% of the EU milk production, estimated at 155 million tons [4]. Environmental and health concerns are shaping the projections for the dairy sector as milk production accounts for a substantial share of overall greenhouse gas emissions (GHGs) in many countries. In the EU, initiatives to adjust dairy technology to reduce such emissions have been implemented, and market analysis indicates that between 2005 and 2025, dairy cattle milk production increased by 53%; however, the decrease in Emission Intensity (EI) limited the increase in total GHGs to 22% [5].

According to FAO Food Balance Sheet data, the average per capita supply of milk and milk products (excl. butter) in EU-27 exceeded 232 kg in 2023, with the highest supply—twice as high—recorded in Denmark (496 kg). In Germany, consumption reached 226 kg, and in Poland, 206 kg. The lowest supply was noted in Cyprus—168 kg and the Czech Republic—176 kg [6]. In both Europe and North America, overall per capita demand for fresh dairy products is declining, but the composition of demand has been shifting over recent years in favour of dairy fat such as full-fat drinking milk and cream. Plant-based dairy replacements are increasingly established and competing more with fresh dairy products than with processed dairy products [1].

During the Anthropocene, defined as the era in which human activity has an overwhelming impact on Earth [7], consumption can be seen as one of the driving forces behind environmental transformation, pollution, and climate change. The impact of increasing demand in many regions is higher than the Earth can sustainably support over the long term. The current food systems, having largely kept pace with population growth, are the single most influential drivers of planetary boundary transgression [8,9]. In the European Union, Luxembourg, Ireland, and Denmark had the highest consumption footprint in 2023, whereas Romania, Slovakia, Bulgaria, and Hungary had the lowest [10]. It has been estimated that halving the consumption of meat, dairy products, and eggs in the region would achieve a 40% reduction in nitrogen emissions, a 25–40% reduction in GHG emissions, and a 23% per capita reduction in the use of cropland for food production [11].

Among the 17 UN SDGs, Target 12.8 calls to ensure that people everywhere have the relevant information and awareness for sustainable development and lifestyles in harmony with nature by 2030 [12]. Target 16 of the Global Biodiversity Framework [13], adopted during the UN Convention on Biological Diversity in 2022, also emphasises the role of access to relevant and accurate information and alternatives on sustainable consumer choices. Based on data that includes socio-cultural aspects of consumer behaviours, supportive policy, as well as legislative or regulatory frameworks, should be established so that the shared vision of living in harmony with nature is fulfilled by 2050 [14].

Although consumer awareness of sustainable food choices in Europe has grown in recent years, driven by environmental concerns and health consciousness, the main barriers to making such decisions remain: availability, cost, and information [15]. Sustainable consumption is a concept that goes beyond the traditional understanding of consumerism and requires responsible purchasing decisions [16]. To move towards the UN SDGs, all people must become more responsible consumers from the environmental and economic perspectives, alongside health and socio-cultural aspects. Such responsibility may benefit from an attitude of openness to change and can be linked to consumer innovativeness, defined as the individual’s tendency or predisposition to buy or adopt products newly introduced in the market, or a preference for a new and different experience [17]. According to previous research, consumers who aim to adhere to the principles of sustainable food production generally show a high acceptance of product innovations if they align with their ethical and environmental values. Innovations that support SDGs are seen not only as technological improvements but also as an expression of the producer’s social responsibility [18,19].

Innovations in the dairy sector, which include new product development, can lead to increased sustainability, replacing well-known products with milk alternatives [20]. The literature shows that food choices and understanding of the concept of sustainability are culture-dependent [21,22]. Innovation is also a cultural phenomenon in which values, norms, and social traditions influence the way new solutions are accepted.

Several publications discussed the overall readiness of consumers to choose sustainable foods and the economic and demographic factors influencing these decisions [23]. Cross-cultural differences in the context of dairy product perceptions have been previously analysed [24]. However, few studies have considered other determinants of choice, such as perceived consumer innovativeness or openness to choosing sustainably produced and novel food. To investigate the impact of such determinants on dairy consumer choices, a survey in Poland and two other EU countries, namely Germany and the Czech Republic, was carried out. This article is structured around a hypothesis that specific consumer concerns about sustainability, especially from the perspective of the natural environment, as well as openness to innovations, motivate consumers to choose sustainably produced foods.

## 2. Materials and Methods

### 2.1. Data Collection Process

Data were collected in December 2024 through an online survey with the use of the Computer-Assisted Web Interviewing (CAWI) technique among adult consumers in three European countries: Poland, Germany, and the Czech Republic. These three countries were selected for the study on the dairy products market due to their similar climatic conditions and well-established dairy production traditions in Western and Central Europe. Moreover, as EU Member States, they are subject to the same quality and safety regulations, ensuring data comparability. Importantly, despite these similarities, the selected countries differ in the maturity of their dairy markets, the availability and market penetration of sustainably produced dairy products, and in consumer purchasing power and environmental awareness. These differences provide a relevant context for analysing how sustainability-related concerns and openness to innovation shape dairy consumer choices across markets at different stages of sustainable food system development.

Altogether, the survey involved 3131 adult respondents, including 1508 (48%) living in Poland, 812 (26%) in Germany, and 811 (26%) in Czechia. Data collection was conducted by a professional market research agency, ARC Rynek i Opinia, which owns a research panel (ePanel.pl) with approximately 50,000 registered users. Panel participants were not drawn from any targeted databases but were recruited online and offline. The response rate on epanel.pl was approximately 20%.

When sending invitations to panellists to participate in the survey, the sample was checked for demographic characteristics, i.e., age, gender, education, and size of place of residence, to ensure that the socio-demographic structure of the groups was representative of the structure of the Polish, Czech, and German populations. However, the selection of respondents from the panel of a specific research agency made it impossible to obtain representative samples in each country, which is a limitation of the study. The Polish sample was selected based on statistical data from the 2021 National Census, including gender, age, education, and place of residence/number of inhabitants in the case of city dwellers. Relevant data were obtained from the Czech Statistical Office (Český Statistický Úřad—2021 data) and the German Federal Statistical Office (Statistisches Bundesamt—2023 data).

The study involved adults (aged 18 and over), both women and men, who use the internet. An additional criterion for inclusion in the survey was the respondent’s declaration at the initial part of the survey, in the so-called screener, that they were responsible for or shared responsibility for deciding on food purchases in the household. In response to the question, ‘Who usually does the grocery shopping in your household?’, the respondent had to choose one of the following answers: only me, I do most of the shopping, or sometimes me and sometimes someone else.

The socio-demographic characteristics of the sample are presented in Table 1.

The study was part of the SUP-RIM project funded by the Polish Ministry of Science and Higher Education. It involved a network of six natural-science universities and aimed to support and advance the Polish dairy sector by conducting scientific research in several interconnected domains, particularly focusing on innovation, product safety and quality, environmental practices, as well as knowledge dissemination.

### 2.2. Ethical Approval

The research was approved on 5 July 2024 by the Rector’s Committee for the Ethics of Research Involving Human Participants of Warsaw University of Life Sciences (protocol no. 31/RKE/2024). Informed consent was obtained at the level of the market research company from all subjects involved in the study.

### 2.3. Description of Questionnaire

The analysis presented in this article was carried out with the use of response data to survey questions linked to sustainability and innovations in the dairy market as determinants of food choices. The self-developed questionnaire, focused on consumer behaviour of European adults, contained questions previously validated by other researchers. The questionnaire was developed based on a review of the relevant literature and previously approved tools. The original version of the questionnaire was prepared in Polish and then translated into German and Czech using a direct and back-translation procedure. The translations were carried out by independent bilingual translators, and discrepancies were resolved by consensus. Prior to data collection, the questionnaire was tested in each country to assess its clarity, cultural adequacy, and internal consistency. Based on the feedback obtained during the tests, minor changes were made to the wording.

Although full psychometric validation was beyond the scope of this study, numerous steps were taken to ensure linguistic equivalence and conceptual consistency across all language versions. Consumer innovativeness with respect to dairy products was measured using the Domain-Specific Innovativeness (DSI) scale, developed by Goldsmith and Hofacker [25]. This scale assesses the degree to which an individual is willing to adopt new innovations in a specific product domain. In our study, the domain was dairy products. Participants responded to six statements from the DSI, three of which have a positive character, referring to openness, curiosity, and willingness to try novel products. The remaining three statements have a negative character, relating to caution, scepticism, or risk avoidance. An example of a positive character item is “If I heard that a novel dairy product was available through a local store, I would be interested enough to buy it”. An example of a negative character item is “Compared to my friends, I seldom buy novel dairy products”.

The sustainability-linked part of our questionnaire refers to the Sustainable Food Choice Questionnaire (SUS-FCQ), developed within the SUSFANS (www.susfans.eu) project. This tool was shown to be a valid and suitable scale to gain a better understanding of the position of sustainability motives against other motives in consumer food choices in multiple countries [26]. SUS-FCQ was validated in multiple countries, including Denmark, the Netherlands, the Czech Republic, Italy, and France. In our study, respondents were asked to what extent they agreed with 7 items, focusing on the general sustainability of dairy products. An example of an item is “My behaviour on the dairy market has no impact on the natural environment”. The questionnaire, translated into the national languages of respondents, was programmed using CADAS software(v. 2024). To verify the quality of the responses, questions were checked for logical consistency, correct completion, and grammatical accuracy. This check included checking the correct operation of filters applied to individual questions, data completeness (whether there was no missing data where responses were required), and logical consistency (whether the answers provided to different questions were mutually exclusive).

### 2.4. Statistical Analysis

A Logistic Regression Model was developed and used to assess the quantitative impact of selected factors on the consumers’ propensity to purchase sustainably produced food. The dependent variable in this model was the degree (on a scale from 1 to 7) to which respondents tried to choose food that is produced sustainably. The model was adjusted for sociodemographic characteristics, such as country of residence, gender, age category, and size of place of residence.

The analysis results obtained were aggregated for the purposes of dichotomizing the dependent variable. Survey participants who selected option 4, “I neither agree nor disagree”, concerning the statement “I try to choose food that is produced in a sustainable way” were classified as “Not/ess interested”. Those adults who declared interest in choosing such food (5–7 on the 1–7 scale) were classified in the “Yes/more interested” category, which was further predicted in the model. Thirteen variables describing attitudes towards product innovation and the level of concern for the natural environment were selected as explanatory variables for the model. As part of the preliminary statistical analyses, the research sample was characterised by the country of study. Next, the variables used in the model were characterised by the category of the dependent variable.

The reason for choosing a dichotomous dependent variable was to predict the willingness to choose sustainably produced food. An alternative could be a model with an ordinal dependent variable. However, the one with a dichotomous dependent variable is more transparent and simpler to interpret the obtained results. In any analysis, the loss of information must be taken into account, but all statistics regarding the predictive accuracy of the model (Likelihood Ratio, Score, and Wald statistic) showed that the explanatory variables used are appropriate predictors of the dependent variable. This was also confirmed by the quite high value of the Max-rescaled R-Square = 0.4321. The predictive correctness of the model was also confirmed by the Hosmer and Lemeshow Test.

All statistical analyses were performed using the SAS 9.4 statistical package at a significance level of α = 0.05. The developed model included variables that were statistically significant for the model at a significance level of α = 0.05. The goodness of fit of the model was assessed using the Likelihood Ratio Test and the Wald Test. The max-rescaled R-Square for this model was 0.435. Besides the Hosmer and Lemeshow test used to assess the accuracy of the predictions, the Association of Predicted Probabilities indicator of the model’s goodness of fit (value of the c statistic) was calculated. Its value c = 0.8401 also confirmed the accuracy of the logistic regression model’s predictions.

## 3. Results

### 3.1. Characteristics of the Surveyed Consumer Sample

The structure of the surveyed sample (N = 3131) of consumers living in Poland, Germany, and Czechia did not differ between countries, with gender (marginally more women than men) and age. The average age among respondents was 43.17 years, with the minimum age being 18 and the maximum age being 70 years (Table 1). The share of adult youth aged 18–24 was around 8% while 25-to-40-year-olds constituted 30.18%. These age groups were of special interest to the authors due to their other research in which young adults’ attitudes were noted as pivotal in driving more sustainable consumption patterns.

The education level and place of residence of respondents from the different countries, however, varied significantly. In the Polish sample, a similar share of respondents (approximately 37%) declared secondary or higher education levels. In Germany, those with secondary education dominated (55%), while in the Czech sample, more than 55% reported having higher education. Considering the place of residence, a bigger share of study participants from Poland lived in rural areas, compared to other countries. In the Czech sample, a statistically higher share of respondents lived in small cities.

### 3.2. Variables Used to Build the Logistic Regression Model

In accordance with the aim of the study for the purpose of statistical analysis, variables describing two main aspects of consumer behaviour were selected, namely the respondents’ innovativeness (referring to their tendency to adopt novel products) and pro-environmental attitudes in choosing dairy products. One category included variables assessing the consumers’ propensity (intention) to purchase novel products, willingness to buy a product that they had never heard of before, and self-assessed knowledge about innovations in the dairy market. The other group of variables used in the analysis reflected the consumers’ environmental awareness and preferences for choosing environmentally friendly products and packaged items (overall beneficial for planetary health) and their belief that their own purchasing decisions impact the natural environment. This group of variables also considered the importance of the product’s place of origin and reducing carbon footprint, the consumer’s propensity to choose products with quality certifications, and perception of the impact on pollution on daily life. All these variables (presented in Table 2) showed statistical significance, which justified their inclusion in constructing the regression model.

### 3.3. Prediction of the Determinants of Food Choices Among Dairy Product Consumers

The quantitative impact of variables on the studied consumers’ interest in choosing sustainably produced food products is indicated by the parameters of the developed Logistic Regression Model (Table 3).

Choosing products produced in an environmentally friendly manner gave an almost twofold increase (94.1%) in the probability of the predicted value of the dependent variable for each point of this opinion (OR: 1.941; 95% CI: 1.71–2.20). Respondents who paid attention to whether products are packaged in an environmentally friendly way were 48% more likely to purchase a product that is produced sustainably (OR: 1.480; 95% CI: 1.32–1.66). Considering the place of origin of dairy products to limit their transport and carbon footprint was linked to a 40.8% increase in the likelihood of choosing sustainably produced dairy (OR: 1.408; 95% CI: 1.27–1.56).

In comparison, the respondents’ awareness of the impact of consumption on planetary health resulted in a 30.6% increase in the likelihood of them choosing sustainably manufactured products for each 1-point increase in this opinion (OR: 1.306; 95% CI: 1.17–1.46). People who chose products with quality certifications were 27.6% more likely to choose products that were produced sustainably (OR: 1.276; 95% CI: 1.14–1.43). Respondents’ awareness of the impact of environmental pollution on their daily lives resulted in a 21% increase in the likelihood of choosing sustainably produced dairy products (OR: 1.210; 95% CI: 1.09–1.34).

Considering purchasing a novel dairy product that the consumer had not heard of before resulted in a 14.8% increase in the likelihood of them purchasing foods produced sustainably for each point of this opinion (OR: 1.148; 95% CI: 1.03–1.27). Each increase (by 1 point) in consumer interest in a novel dairy product available in stores had a stimulating effect on the dependent variable, resulting in a 12.3% greater chance of choosing sustainably produced foods (OR: 1.123; 95% CI: 1.01–1.25). Those respondents who believed that their behaviour on the dairy market has no impact on the environment were 6.4% less likely to choose products manufactured in a sustainable way (OR: 0.936; 95% CI: 0.85–0.99).

The conducted analysis also indicated that among all the variables selected for the Regression Model adjustment, only the country of residence proved to be statistically significant. German consumers were 33.8% more likely to choose sustainably produced products than Polish consumers (OR: 1.338; 95% CI: 1.08–1.66), while Czech consumers were 10% less likely to do so compared to Polish consumers (OR: 0.900; 95% CI: 0.73–0.98).

## 4. Discussion

In light of our study, the two variables that had the most stimulating effect on sustainable consumer choices in the EU dairy market were linked to the way in which food products are manufactured and/or packaged. A similar observation was made by [27], who demonstrated that environmental awareness is the biggest predictor of consumer intention to purchase sustainably produced foods. According to the theory of planned behaviour [28], attitudes towards a particular behaviour—in this case, choosing food produced and packaged with respect to the natural environment—play a key role in creating readiness to act responsibly. Research conducted by Taufique and Vaithianathan [29] as well as Jakubowska et al. [30], also confirmed that positive attitudes towards eco-friendly products increase consumers’ intention to purchase them, especially among younger individuals. This study also shows that, in the case of Polish Generation Z consumers, knowledge is an important predictor of sustainable consumption, in addition to attitudes. A study presenting a meta-analysis of 41 peer-reviewed publications from 2008 to 2023 [31] showed that past experience was the strongest predictor of sustainable consumer behaviour. It means that consumers who have previously used sustainable products are more likely to continue doing so. Factors such as knowledge, perceived behavioural control, and environmental consciousness also showed a significant impact, highlighting the importance of awareness and perceived agency in encouraging sustainable consumption. Previous studies on factors influencing consumer behaviours in EU countries show that consumers are also becoming increasingly concerned about the harmful effects of packaging waste on the environment. In several studies, packaging has been highlighted as a major source of non-biodegradable waste that harms the environment [32,33]. A recent Italian study indicated that the intention to purchase milk in biodegradable packaging is still low, as consumers declare low levels of willingness to pay for milk offered in biodegradable packaging, regardless of the raw material used [34].

The third strongest factor that was identified in our analysis to drive the probability of choosing food produced sustainably was taking into account the place of origin of dairy products to limit their transport and carbon footprint. This seems to be a rather false stereotype in perceiving transport as the main culprit of carbon dioxide air pollution. IPCC data indicate that, on a global scale, transport is the fourth largest emitter of greenhouse gases (15%) among the main types of economic activity (the top spots are occupied by electricity and heat production, industry, and agriculture, forestry, and other land use) [35]. An Australian study also reported that consumers overstate the effects of transport, particularly air-freighted food, on the environment [36]. However, transportation plays a crucial role in the life cycle of perishable fresh dairy items that require controlled temperature conditions [37]. Promoting local and seasonal products and optimising supply chains can reduce negative environmental impacts and contribute to territorial economic growth [38]. Territoriality has also been confirmed to be perceived as an index of higher product quality [39].

Awareness of the relationship between food consumption and planetary health, as well as the belief that environmental pollution affects their daily lives, also influenced sustainable food choices among the study participants. Thus, pro-environmental attitudes apply to food consumption in general, not just dairy products. Issues related to the transformation of food systems and changes in their consumption stage have finally entered the public debate and the activities of governments, producers, scientists, and other stakeholders. That is why awareness of how food consumption and individual food choices impact planetary health is growing [40,41]. However, research shows that the declarative awareness of the impact of food consumption on the environment and the impact of environmental pollution on aspects of everyday life is at the level of generality and does not apply to all the principles of a sustainable diet [42,43,44].

Scientific literature has shown that an attitude–behaviour gap exists between the declared readiness and the act of purchase, linked to economic barriers, limited availability of products, or lack of trust in certificates [45]. In our research, choosing certified food products turned out to be one of the weakest variables driving sustainable food choices.

Distrust of certificates may stem from their enormous number and diversity, leading to consumer confusion, a lack of label uniformity, greenwashing, limited visibility and availability at points of sale, consumer price sensitivity, and the prioritisation of other factors [46]. The perception of certificates and their use in food choices is influenced by many factors, such as the age, gender, and education of consumers, as well as the geographical market, market infrastructure, and marketing. In a survey of Italian consumers [47], women showed significantly higher levels of knowledge and placed more importance on specific sustainable certifications. Younger consumers demonstrated a profound knowledge of environmentally focused certifications, such as organic certificates, the carbon footprint, and the FSC (Forest Stewardship Council). The low importance of certifications as a factor in sustainable food choices may be related to a limited understanding of certificates and certification systems. This suggests that while consumers may recognise labels, deeper educational efforts are needed to increase understanding and trust in these certifications [47]. Knowledge is the basis for purchasing certified food and is a factor of freedom in the decision-making process, but purchases are also determined by consumers’ tight budget constraints. Organic food and food labelled with other certifications generally have higher prices, which constitute an economic barrier to purchase. Economic factors in food choice have become more important in recent years. The results of a survey coordinated by the European Commission [48] show that cost (60%) is the most frequently cited factor influencing food purchases among EU citizens. The weight of the economic factor has risen dramatically, by six points compared to 2022, and is decisive in twenty Member States. Among them are three countries covered by our survey, with 76% of respondents from the Czech Republic, 62% from Germany, and 60% from Poland. Furthermore, the survey revealed that the perceptions of the link between human health and environmental and plant-related issues have shifted from “strong impact” to “moderate impact”. The constraints on the influence of certification as a factor in choosing sustainable food, as revealed in both our survey and the European survey, indicate a need to mitigate them. To this end, regular and frequent educational and information initiatives should be undertaken, and universal and unambiguous food labelling regulations should be introduced, highlighting the significant role of certifications in promoting sustainable food consumption. For this purpose, it is also proposed to introduce standardised international criteria in order to improve transparency and meet consumer expectations [49].

The drive to transform food systems into sustainable ones will largely depend on consumer behaviour and the popularity of plant-based diets. Based on an integrative literature review (ILR) prepared by [50], it was concluded that people and their behaviour are central to the current transformation challenges, i.e., dietary shifts, changes in consumer behaviour, reducing food waste, and policy reform.

Our research results additionally proved that the innovativeness of European consumers drives their attempts to buy sustainably produced foods. In this study, innovativeness is understood as a multidimensional concept encompassing behavioural openness to new products, acceptance of technological solutions in food production, and attitude towards innovations.

The strongest variable in this group was willingness to buy novel dairy products, even if the potential buyer had never heard of them before, which reflects the behavioural dimension of innovativeness related to consumers’ readiness to experiment and reduce uncertainty in purchasing decisions.

The fact that openness to technological innovations determines the acceptance of more sustainably produced foods was also observed in other countries, including Australia, Singapore, and the USA [19], highlighting the technological dimension of innovativeness associated with trust in new production methods and processes. According to research conducted in EU Member States, such as Germany and the Czech Republic, consumers focus more on quality and environmental aspects rather than on the novelty itself [51], suggesting that product innovativeness is more readily accepted when it is perceived as improving functional or sustainability-related attributes rather than novelty per se. Educational activities can support the development of responsible consumer behaviours and increase the trust in novel products [19,52], thereby reinforcing behavioural innovativeness. From a technological perspective, innovations (e.g., extraction of plant-based proteins) have enabled producers to develop a wide variety of dairy alternatives [53].

Another systematic review proved that environmentally friendly consumers are more willing to experiment with novel product categories, especially if innovations are aimed at increasing sustainability and measurable environmental benefits [54]. Factors that strengthened the acceptance of novel food products included not only the environmental awareness of consumers, but also their perception of food safety and compliance with ethical values [55]. Additionally, Günden [56] observed that novel foods were more positively assessed if they combined the elements of novelty with functionality and social and environmental benefits, which further underlines the interconnection between product, technological, and behavioural dimensions of innovativeness.

Another finding of our study is that those consumers who stated that their behaviour on the dairy market has no impact on the natural environment declared making weaker attempts to choose food produced sustainably. This can be interpreted in the context of empowerment theories that connect individual well-being with the larger social environment and suggest that people need opportunities to become active in community decision-making to improve their lives [57]. From this perspective, behavioural innovativeness may also depend on consumers’ perceived belief in the effectiveness of their choices. The EU Consumer Policy, which currently aims at empowering consumers to make their lifestyles more sustainable, could potentially play a role in supporting this process and make consumers feel more in control [58]. However, in the era of EU discontent, rethinking consumer empowerment seems to be essential to setting new directions for more sustainable consumer choices [59].

Of all the demographic variables, only the country of residence proved to be significant in driving the consumers’ choice of sustainably produced foods. Compared to Polish consumers, German residents were more likely to choose such products, while Czech consumers were less likely to do so. Similarly, it was previously observed that cultural aspects can both accelerate and halt the process of implementing innovations. In societies open to change, consumers are more likely to choose novel products, such as plant-based milk alternatives. On the other hand, in more traditional societies, innovative products need to be adapted to local norms and consumption patterns to be accepted. Introducing more sustainable plant-based alternatives requires cultural adaptation by assigning them meanings related to health, planetary care, or a modern lifestyle [20].

Our study provides valuable insights into sustainable food choices, with a particular focus on innovative dairy products. However, we are aware of certain limitations of this study. The use of the quota selection method ensured that each sample was representative of the demographic and social structure of the population in each country. However, the selection of respondents from the specific research panel constituted a limitation of the study in terms of representativeness at the national level. For example, the group of German consumers was too small in relation to the number of citizens in that country. Additionally, the samples differed significantly in terms of education level and place of residence. Furthermore, the study allowed us to learn about behavioural intentions rather than purchasing behaviour itself. The purchasing decision-making process at points of sale is determined by a combination of various additional factors, including economic ones, which limit choices. Although the spatial scope of the study covered three neighbouring countries (each with each other) in Central Europe, there are some cultural differences between them. These may also influence choices and behaviours related to sustainable food consumption.

The issue of the origin of food products (e.g., country of origin—COO) had not been analysed in our study and could be potentially taken up in future research, especially if non-EU Member States are compared. Future research should also consider other combinations of factors, including animal welfare, more equitable and affordable access to foods, etc., that will allow for a more holistic approach and a deeper understanding of consumer choices. Generational differences are also worth addressing in future studies, as in times of rapid technological development and socio-cultural changes, such differences are becoming more pronounced and determine different strategies to encourage more sustainable food consumption in families with children, school settings, etc.

## 5. Conclusions

The aim of our study was to examine whether environmental issues and product innovation in the dairy market influence Europeans from three countries—Poland, the Czech Republic, and Germany—in their choice of sustainable food. The use of statements from the Sustainable Food Choice Questionnaire (SUS-FCQ) and the Domain-Specific Innovativeness Scale (DSI) is an approach that allowed for the construction of the Logistic Regression Model to analyse how consumer attitudes towards sustainability and innovations are linked to behaviours.

In light of our research, both environmental concerns and the innovativeness of dairy consumers living in three EU countries go hand in hand in driving their sustainable food choices. The conducted statistical analysis has shown that among the studied variables, environmental concerns, mainly linked to sustainable production, packaging, and transport, have the strongest impact on consumer behaviours. Awareness of the impact of food consumption on the planetary environment and product labelling with certification symbols was of lesser importance. The results show the need, and even the necessity, to shape positive consumer attitudes and provide widespread access to various forms of education in the area of mutual interactions between the environment, food choices, and the health of the population. Targeted information and educational campaigns (also on social media, especially to younger generations) can be used as tools for disseminating knowledge about sustainable consumption and production. Our findings also provide valuable evidence for policymakers, the business community, and educators to ensure that sustainable food consumption becomes part of a comprehensive strategy to improve the health of people and the planet. Since there is considerable scientific evidence that some consumers are wary of novel foods and associated novel food technologies [60,61], educating consumers about sustainability-focused innovations in the dairy sector would support further efforts to reduce the environmental burden of agri-food systems.

## Figures and Tables

**Table 1:** Socio-demographic characteristics of the studied sample.

Variables	Total Sample N = 3131 (100%)	Polish N = 1508 (100%)	German N = 812 (100%)	Czech N = 811 (100%)	*p*-Value ^#^
Gender					
Women	1623 (51.84)	794 (52.65)	404 (49.75)	425 (52.40)	0.3833
Men	1508 (48.16)	714 (47.35)	408 (50.25)	386 (47.60)	
Age (years)					
18–24	252 (8.05)	127 (8.42)	57 (7.02)	68 (8.38)	0.154
25–40	945 (30.18)	467 (30.97)	223 (27.46)	255 (31.44)	
41+ Mean (Median)	1934 (61.77) 43.17 (43.00)	914 (60.61) 42.33 (41.00)	532 (65.52) 44.98 (45,50)	488 (60.18) 42.92 (42.92)	
Education					
Primary/Vocational	632 (20.19)	386 (25.60)	156 (19.21)	90 (11.10)	<0.0001
Secondary	1290 (41.20)	564 (37.40)	452 (55.67)	274 (33.78)	
Higher	1209 (38.61)	558 (37.00)	204 (25.12)	447 (55.12)	
Place of residence					
Rural area	841 (26.85)	502 (33.29)	171 (21.06)	168 (20.72)	<0.0001
City–inhabitants Less than 20,000	561 (17.92)	185 (12.27)	151 (18.60)	225 (27.74)	
20,000–50,000	467 (14.92)	197 (13.06)	139 (17.12)	131 (16.15)	
50,000–200,000	565 (18.05)	303 (20.09)	144 (17.73)	118 (14.55)	
More than 200,000	697 (22.26)	321 (21.29)	207 (25.49)	169 (20.84)	

^#^ Significance of the Chi-square test.

**Table 2:** Characteristics of the independent variables used to build the Logistic Regression Model in accordance with the dependent variable.

Variable	No (N = 1450) ^#^	Yes (N = 1681) ^#^	*p* ^##^	Min	Max	Median
In general, I am among the last in my circle of friends to purchase a novel dairy product	1519.2	1606.4	0.0025	1	5	3
If I heard that a novel dairy product was available through a local store, I would be interested enough to buy it	1362.4	1741.6	<0.0001	1	5	3
Compared to my friends, I seldom shop for novel dairy products	1541.0	1587.6	0.0662	1	5	3
I would consider buying a novel dairy product even if I hadn’t heard of it yet	1399.4	1709.7	<0.0001	1	5	3
In general, I am the last in my circle of friends to know the names of the latest dairy product and dairy product trends	1540.0	1588.5	0.0584	1	5	3
I know more about novel dairy products than other people do	1364.3	1740.0	<0.0001	1	5	3
I choose dairy products that are produced in an environmentally friendly way	1095.2	1972.1	<0.0001	1	5	3
I pay attention if the product is packaged in an environmentally friendly way	1119.2	1951.4	<0.0001	1	5	3
My behaviour on the dairy market has no impact on the natural environment	1633.0	1508.2	<0.0001	1	5	3
I consider the place of origin of dairy products to limit their transport and carbon footprint	1144.8	1928.3	<0.0001	1	5	3
I choose products with a quality certificate	1212.2	1871.3	<0.0001	1	5	3
Environmental pollution impacts my daily life	1238.6	1848.4	<0.0001	1	5	4
I am aware of food consumption’s impact on planetary health	1244.8	1843.0	<0.0001	1	5	4

^#^ Dependent variable level (predicted level—“YES”) ^##^ *p*-value U Mann–Whitney Test.

**Table 3:** Parameters of the Logistic Regression Model for Sustainable Food Choice.

Parameter	Level ^#^	Estimate ^##^	Point Estimate ^###^	95% Wald Confidence Limits		Standard Error	Wald Chi-Square	*p*-Value
Intercept		−7.542				0.37	417.5	<0.0001
I choose dairy products that are produced in an environmentally friendly way		0.663	1.941	1.71	2.20	0.06	104.86	<0.0001
I pay attention if the product is packaged in an environmentally friendly way		0.392	1.480	1.32	1.66	0.06	46.42	<0.0001
I consider the place of origin of dairy products to limit their transport and carbon footprint		0.342	1.408	1.27	1.56	0.05	42.99	<0.0001
I am aware of food consumption’s impact on planetary health		0.267	1.306	1.17	1.46	0.06	22.67	<0.0001
I choose products with quality certificates		0.244	1.276	1.14	1.43	0.06	18.62	<0.0001
Environmental pollution impacts my daily life		0.191	1.210	1.09	1.34	0.05	13.06	0.0003
I would consider buying a novel dairy product even if I hadn’t heard of it yet		0.138	1.148	1.03	1.27	0.05	6.75	0.0094
If I heard that a novel dairy product was available through a local store, I would be interested enough to buy it		0.116	1.123	1.01	1.25	0.05	4.33	0.0373
My behaviour on the dairy market has no impact on the natural environment		−0.066	0.936	0.85	0.99	0.05	1.86	0.0472
Country	Germany	0.291	1.338	1.08	1.66	0.11	7.03	0.0080
	Czech Republic	−0.105	0.900	0.73	0.98	0.11	0.93	0.0435
	Poland (ref.)	0	1					

Only variables that are statistically significant at the level α = 0.05 were included in the model. ^#^ Level—qualitative variable level; ^##^ Estimate—β parameter; ^###^ Point estimate—e^β^ (ODS ratio).

## Data Availability

The original contributions presented in the study are included in the article. Further inquiries can be directed to the corresponding authors.
